# Osseous cystic echinococcosis: A case series study at a referral unit in Spain

**DOI:** 10.1371/journal.pntd.0007006

**Published:** 2019-02-19

**Authors:** Begoña Monge-Maillo, María Olmedo Samperio, José Antonio Pérez-Molina, Francesca Norman, Carla Ruth Mejía, Sandra Chamorro Tojeiro, Rogelio López-Vélez

**Affiliations:** National Referral Unit of Tropical Diseases, Infectious Diseases Department, Ramón y Cajal University Hospital, IRICYS, Madrid, Spain; Istituto Superiore di Sanità, ITALY

## Abstract

**Background:**

Cystic echinococcosis (CE) is present in all continents, except for the Antarctica. Characteristically, CE lesions are found in the liver and the lungs, but virtually any part of the body may be affected (the spleen, kidneys, heart, central nervous system, bones, among others). It is estimated that the incidence of bone involvement in CE is 0.5% to 4%.

**Methodology:**

A retrospective study was performed of patients with osseous CE treated at the National Reference Unit of Tropical Diseases of the Ramon y Cajal Hospital, Madrid, Spain, between 1989 and December 2017. Epidemiological, clinical, diagnostic and therapeutic data of patients with long-term follow-up were collected.

**Main findings:**

During the study period, of the 104 patients with CE, 27 exhibited bone involvement (26%). The bones most frequently affected were the spine, followed by the ribs, pelvis, femur, tibia and the scapula. The most common symptom was pain followed by medullar syndrome and pathologic fracture. In total, 81.5% of patients underwent surgery for osseous CE at least once. As many as 96% received albendazol either in (mostly long-term) monotherapy or in combination with praziquantel.

**Conclusions:**

The diagnosis and management of osseous CE is challenging. In many cases osseous CE should be considered a chronic disease and should be managed on a case-by-case basis. Lifelong follow-up should be performed for potential recurrence and sequels.

## Author summary

Echinococcosis occurs in humans as a result of infection by a cestodes of the genus *Echinococcus*. One of the species, *E*. *granulosus*, causes cystic echinococcosis (CE) in humans worldwide. In the lifecycle there is a definitive host (generally dogs) which host this parasites at the small bowel. From there, ground is shed with the eggs of the parasite through feces and the intermediate host (usually a sheep or other herbivores get infected). Humans act as an incidental intermediate host when they become infected through the consumption of water or food contaminated with *Echinococcus* eggs. Once the egg has been ingested, it penetrates the intestinal mucosa and through the circulatory system finds an anatomical site forming a cystic lesion (hydatid or hydatid cyst). Characteristically, CE are found in the liver and the lungs, but virtually any part of the body may be affected. Incidence of osseous CE is low, its diagnosis and management is challenging and there is little information published. In this study we report our experience at a referral unit during nearly 30 years in the management of a series of patients with osseous CE. Such information may be useful for other physicians when treating osseous CE.

## Introduction

Echinococcosis occurs in humans as a result of infection by the larval stages of cestodes of the genus *Echinococcus* [1]. Four species pose a risk to human health, namely: *E*. *granulosus* species complex, (which is subdivided into *E*. *granulosus* sensu stricto, *Echinococcus felidis*, *Echinococcus equinus*, *Echinococcus ortleppi* and *Echinococcus canadensis*) which causes cystic echinococcosis (CE) and occurs worldwide including tropical and subtropical regions [2]; *E*. *multilocularis*, which causes alveolar echinococcosis and is confined to the northern hemisphere; and *E*. *vogeli* which cause neotropical polycystic echinococcosis and *E*. *oligarthrus* which cause neotropical unicystic echinococcosis that only occur in Latin America. Several studies have shown that Echinococcosis present an increasing risk to public health and can be regarded as an emerging or re-emerging disease [3].

In CE, the lifecycle of the parasite involves two hosts: a) the definitive host–generally dogs, although other carnivores such as wolves, dingoes, hyenas can also host this parasite. Adult parasites attach to the mucosa of the small bowel through hooklets and suckers and, from there, ground is shed with the eggs of the parasite through feces. b) The intermediate host–usually a sheep or other herbivores such as goats, horses, camels or pigs, among others–gets infected by the ingestion of ground contaminated with the eggs of the parasite. Once the egg has been ingested, the embryo hatches and penetrates the intestinal mucosa, enters host’s circulatory system and develops in the vesicular metacestode when it finds a suitable anatomical site. This stage of the parasite is a unilocular, fluid-filled cystic lesion (hydatid or hydatid cyst). When the definitive host eats the viscera with the hydatid cyst, the cycle is completed. Humans act as an incidental intermediate host when they become infected with oncospheres through the consumption of water or food contaminated with *Echinococcus* eggs [4].

Cystic echinococcosis is present in all continents except for the Antarctica. It primarily occurs in the Mediterranean basin, the Middle East, central Asia, western China, the Russian Federation, Latin America and north and east Africa. The prevalence of CE may exceed 5%, with incidence rates of 50/100 000 person-year in some areas such as South America (mainly Peru and Argentina), east Africa (mainly Kenya) and Asia (mainly China) [3, 5].

In Spain, only infections with *E*. *granulosus* have been identified. Human CE was a mandatory notifiable disease from 1982 to 1996, being an important anthropo-zoonosis in terms of incidence and morbidity [6]. In 1985, epidemiological data showed an incidence of CE of 2.5/100,000 per year, with nearly 1000 new cases every year. The incidence of CE progressively decreased from 600–700 new cases per year in the 1980s to 300–500 new cases/year in the 1990s. In 1997, the incidence of CE was 0.78/100,000 per year. This decrease was probably the result of national control programs mainly based on slaughterhouse hygiene, public education and the regular administration of praziquantel to dogs [7]. However, incidence rates might be underestimated. The reason is that CE stopped being a mandatory notifiable disease in 1996 and, since then, surveillance has been primarily carried out in the autonomous communities where CE is endemic. Underestimation of incidence was shown in an epidemiological study of CE in Spain in the 1997–2012 period based on data from a Centralized Hospital Discharge Database. Incidence rates were found to be higher than the ones reported in previous studies [8].

Characteristically, CE lesions are found in the liver (70%) and the lungs (20%), but virtually any part of the body may be affected (the spleen, kidneys, heart, central nervous system, or bones). Based on published data, it is estimated that the incidence of bone involvement in CE is 0.5% to 4% of all cases of CE [5]. Sixty per cent of cases of osseous CE have been reported in Europe (especially in Turkey, Germany and Spain) and the former Soviet Union [6]. CE usually affects a single bone and the most frequent site of bone lesions is the vertebral column (40%-50%), followed by large bones (25%-30%), the pelvis (15%-20%) and–less frequently–the cranium, sternum, scapula and the phalanges [9,10].

Published literature on the management of osseous CE–a rare manifestation of a neglected disease–is scarce. This article describes the symptoms and management of patients with osseous CE treated in the National Referral Unit of Tropical Diseases in Madrid with long follow-up and compare the results obtained with the ones reported in the literature. The objective of this study is to increase the knowledge on the management and evolution of this neglected tropical disease and to be of use a reference for other physician who have to manage patients with osseous CE.

## Methods

Ethics statement: As this is a retrospective analysis, data were analyzed anonymously, and written informed consent was not required from patients. The database from which patients’ information was obtained was approved by the Ramón y Cajal Hospital’s Ethics Committee (Comité Ético de Investigación Clínica, CEIC, Hospital Ramón y Cajal) and was used in accordance with current laws in Spain (Ley Orgánica de Protección de Datos de Carácter Personal 15/1999) that guarantee personal data protection.

A retrospective study was performed of patients with osseous CE treated in the National Referral Unit of Tropical Diseases of Ramon y Cajal Hospital of Madrid, Spain between 1989 and December 2017. The data collected included: a) Epidemiological data: age at diagnosis, gender and epidemiological risk factors for CE infection; b) Characteristics of osseous CE: location–categorized as axial skeleton (spinal, ribs and pelvis CE), appendicular skeleton (only limbs) and others–; number of sites involved, and evidence of concomitant CE in other visceral or soft tissues. The Dew/Braithwaite & Lees classification was not included to describe the spinal CE cases because it was not considerate necessary for the purpose of this study. c) Clinical presentation by bone location. d) Diagnostic variables: how the disease was first diagnosed, specific serological test, and the presence (or absence) of eosinophilia. e) Treatment: antiparasitic treatment (drugs administered, duration and toxicity of treatment), surgical treatment and percutaneous treatment. f) Follow-up: mean follow-up, complications after surgery, clinical outcome and sequela.

## Results

A total of 104 cases of CE were managed during the study period, of which 27 corresponded to osseous CE (26%).

### Epidemiological data

-Sixteen patients were male (59.2%).

-The average age at diagnosis was 36 (IQR 24–62 years) being three patients under 20 years-old.

-All patients had lived in rural regions of Spain for a long time. Yet, exposure was limited to childhood in a case and to summer vacations in the second.

### Characteristics of osseous CE

-In total, 44 bone sites were identified in the 27 patients with osseous CE.

**Fig 1:**
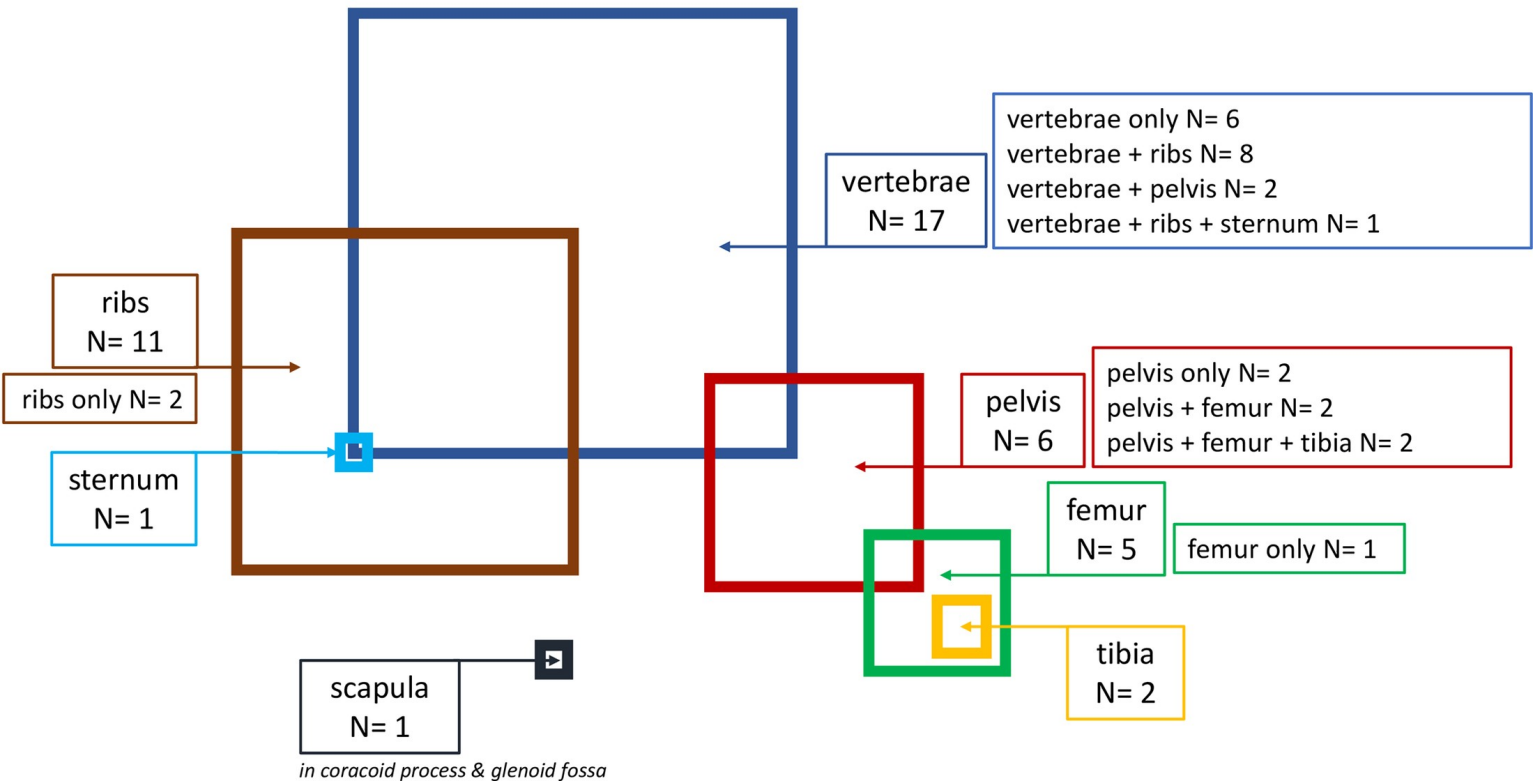
Summary of sites of osseous cystic echinococcosis: Total: 44 sites in 27 patients; Scapula: In coracoid process & glenoid fossa.

-Twelve patients had only a bone involved; another 12 patients had two bones affected, and three patients had three or more bones involved. Therefore, more than half of patients had two or more bones affected by CE. The bones most frequently involved were the spine (17), followed by the ribs (11), pelvis (6), femur (5), tibia (2) and the scapula (1) (Fig 1).

**Fig 2:**
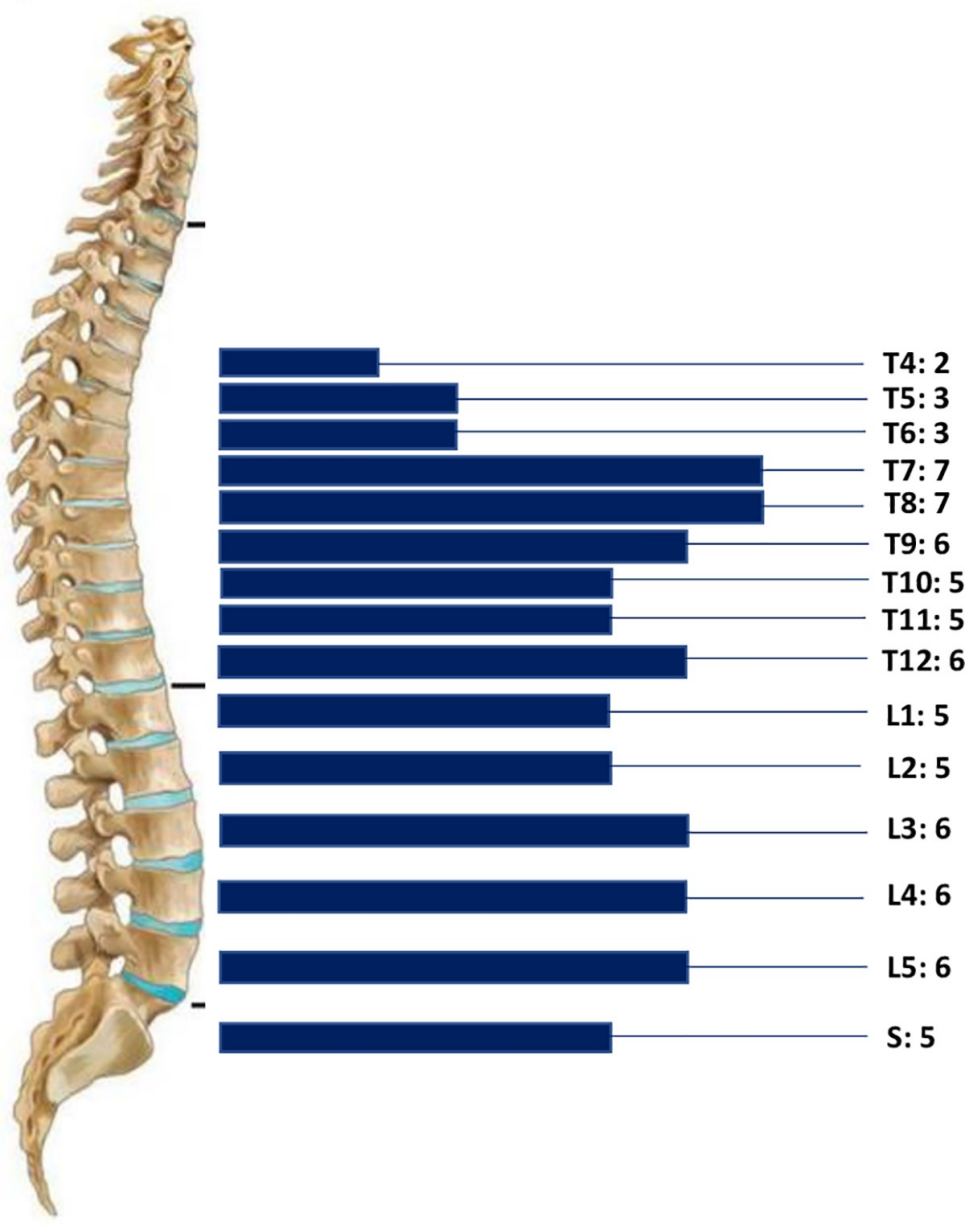
Distribution of the individual vertebral levels involved.

- In the 17 patients with spinal osseous CE, lesions were located in the thoracic spine in eight patients (47%), the thoraco-lumbar spine in three (17.7%), the lumbo-sacrum spine in three (17.7%), the lumbar spine in one (5.8%), the sacrum in one (5.8%), and the thoraco-lumbo-sacral spine in a patient (5.8%).(Fig 2).

-Twelve of the patients had concomitant extraosseous CE: four in the lungs, two in the liver and six in both, the lung and the liver.

### Clinical presentation by bone location

-The most frequent clinical presentation was pain, which was reported in 16 patients (59.2%): seven located in the spine, six in the pelvis, two in the ribs and one in the scapula, followed by medullar syndrome in 10 patients (37%) all located in the spine. A patient had a pathologic fracture (3.7%) in the femur.

### Diagnostic variables

-The disease was first diagnosed by radiology (plain X-ray, computed tomography or magnetic resonance) in 18 patients (66.7%), surgery on suspicion of another illness in seven patients (26%), or by a biopsy of the lesion on suspicion of a bone tumor in two patients (7.4%).

-Serology (IHA followed by ELISA) was performed in 17 patients, with heterogeneity of the serology tests employed a long the study period, with 10 (59%) positive results.

-A hemogram was performed to all the patients. Six patients were positive for eosinophilia (22.2%).

### Treatment characteristics

-Antiparasitic treatment: of the 27 patients included, 26 received antiparasitic therapy with albendazole 400mg/12 hours. A total of 18 received continuous long-term treatment with albendazole for an average of six years (IQR 0.25 to 16 years). Another seven patients received albendazole discontinuously for long periods. In a patient, treatment with albendazole was interrupted due to side effects. Sixteen (59.3%) patients were administered a combination therapy of albendazole plus praziquantel 600mg/8 hours for an average of five years (IQR 4 months to 15 years). Five patients with spinal involvement received a combination therapy with albendazole plus praziquantel plus nitazoxanide 500mg/12 hours. The duration of this combination therapy was four years in two patients and one year in a patient. Two patients were lost to follow-up and no data were available on the duration of treatment. Despite the long duration of antiparasitic therapy, tolerability was good. In fact, most patients had received antiparasitic drugs (Albendazole +/- Praziquantel) for more than 10 years on average with no side effects. A slight increase in transaminases was reported in seven patients, although treatment discontinuance was not required. Treatment had to be stopped due to side effects in only three patients: a patient developed alopecia and oral ulcers due to albendazole; another patient manifested angioedema under praziquantel treatment; and a patient developed exanthema during nitaxozanide treatment.

-Surgery: Twenty-two patients (81.5%) underwent at least a surgical intervention for osseous CE, none of which was curative. The average number of surgical interventions per patient for osseous CE by body site was: 10.2 (ranging from none to 32 interventions per patient) times for pelvic CE; 3.95 (ranging from 1 to 10 interventions per patient) times for spinal CE, 2.00 times for femur CE, and 1.00 time for rib CE (Table 1). In patients with spinal CE, the most common intervention was laminectomy. Eight patients needed surgical fixation. Total limb amputation was performed in two of the six patients with pelvic CE, and another two needed iliac osteotomy. Patients with femur CE required bone resection and prosthesis.

**Table 1:** Number of interventions and complications of the interventions.

Skeletal area	Location	Num patients	Num intervetions (average)	Num interventions (range)	Complications	Num patients
Axial	Vertebrae	17	3.95	1 to 10	Infection	10
					Fisures	6
					Fixation material broken	1
					Iliac thrombosis	1
					Death	1
	Pelvis	6	10.2	0 to 32	Infection	3
					Fistule	2
					Phantom limb pain	1
	Ribs	2	1	n/a	Fistule	1
Limbs	Femur	1	2	n/a	No complication	0
	Scapula	1	0	n/a	No complication	0

n/a: not applicable

-Percutaneous treatment: the PAIR technique (Puncture, Aspiration, Injection and Re-aspiration) was used in four patients. In three cases, puncture was in patients with CE in soft tissues next to osseous CE sites (two in the spine and one in the ribs). In the fourth patient, PAIR was performed for spinal decompression. Initially, neurological symptoms improved, but the patient ultimately relapsed.

### Follow-up

-The average duration of follow-up was more than 20 years. Distribution of duration of follow up was: < 5 years follow up: 4 patients; 5–10 years follow up: 1 patient; 10–20 years follow up: 7 patients; >20 years follow up: 15 patients.

-Twenty-six episodes of complications associated with surgery were reported, being secondary bacterial infection the most frequent (16). A patient had the fixation material broken, another had iliac thrombosis and one died due to probable infectious abscess in the surgical area and respiratory infection three months after surgery (Table 1).

-The most frequent sequels in spinal CE was medullary syndrome (13 patients) and severe pain (3 patients). Two patients with pelvic CE had a limb amputated and another patient developed severe functional disability. In patients with rib CE and femur CE, the most frequent sequel was severe residual pain.

-Despite surgery and long-term antiparasitic treatment, 25 of the 27 (92.6%) patients ultimately relapsed. Recurrence was not reported in two patients. Yet, these patients had been recently diagnosed patients and only had one-year follow-up.

## Discussion

Osseous CE is a rare location of hydatid diseases. In fact, 29 years of study were needed to get 27 cases. However, the prevalence of osseous CE among all cases of hydatid disease in this study was much higher than the one reported in the literature (26% vs 0.5%-4%) [11]. This may be due to the fact that our center is a referral unit of tropical and parasitological diseases, where less frequent o more complicated infectious diseases are managed.

The average age at diagnosis in our sample was 36 years mostly affecting men. This is consistent with the results of other series, where osseous CE was found to mostly occur in immunocompetent men with a median age of 37 years (peak age of disease is 21–40 years) [10,12].

According to the literature, osseous CE prevailingly invades the spine (45%), pelvis (14%), femur (10%), ribs (8%), humerus (2%), and less frequently other sites as the cranium, sternum, scapula and the phalanges [10,11,13,14]. This supports our results, except for the fact that, in our study, the ribs were more frequently affected than the pelvis or the femur, being rib involvement generally associated with spine involvement. As reported in the literature, in our series multifocal osseous involvement was prevailingly observed in patients with spinal echinococcosis, being the thoracic and lumbar spine the sites most frequently involved [1]. In fact, when several bones are affected, spread usually occurs by direct propagation, which would explain that most multifocal osseous sites were associated with spinal and rib involvement.

Osseous CE can remain asymptomatic for long due to its slow growth inside the bones. When symptoms appear, pain is the most frequent followed by pathological fracture, functional impairment or local swelling. Based on our data, nearly 60% of patients presented with pain. However, when the spine is involved, the most common symptoms are those associated with spinal cord compression or vertebral bone destruction [1] as it was observed in our series, where medullar syndrome was the second most frequent symptom. The symptom of spine CE may vary depending on the localization of the cystic which has been classified on five types (Dew/Braithwaite & Lees classification): type 1, medullar; type 2, intradural; type 3, extradural; type 4, vertebral; and type 5, paravertebral [15,16].

In most of our cases, diagnosis was performed by radiology with computed tomography or magnetic resonance. In patients with epidemiological risk factors, radiological findings are the most common initial signs suggestive of osseous CE. However, differential diagnosis with bone tumor, infection or inflammatory disease is required. Diagnosis based on radiological scanning is challenging, as there are no specific radiological findings for osseous CE. Generally, the most common findings are a single or multiple extensive osteolytic lesion containing trabeculae with cortical thinning. In other cases, pathological fractures with a periosteal reaction or lesions with calcification in neighboring soft tissues due to proximal spread can also be found [17,18]. In the spine, lesions can be described as a “bunch of grapes”. In this case, it is important to determine if there is neurological involvement or not. Initially, lesions are located at the vertebral body, but it can affect the canal, the perirachial space, the ribs or neighboring vertebrae [19].

Serology was performed in 63% of patients, although different testing techniques were used over the long period of study. This is important, because the sensitivity of serology can vary depending on the serological test employed [20–23]. Yet, the sensitivity of serology also depends on the integrity of CE and its location. In osseous CE, serology may be more sensitive due to the absence of a fibrous capsule and the contiguity of cyst CE to the tissues. Yet, the specific sensitivity of serology in these cases is unknown. In our series, we obtained a 60% sensitivity (taking into account the heterogeneity of the serology tests employed and the localization of osseous CE).

In some cases, uncertainty about diagnosis and the urgent need for differential diagnosis with bone tumors led to a surgical procedure for confirmation of diagnosis. However, except for cases where surgery is a therapeutic option, surgery and also the aspiration of the cyst must be avoided due to the risk for potential local dissemination, sensitization or even anaphylaxis [24]. Unfortunately, in many cases this cannot be avoided. In our series, 26% of patients underwent surgery on suspicion of another illness, and aspiration for anatomopathological diagnosis was performed in 7.4%.

Regarding therapeutic options, we found that more than 80% of patients underwent surgery, although complete curative results were not achieved. It is known that the only real curative approach for osseous CE is radical resection surgery. Yet, this is rarely possible, especially when the axial bone-spine, the pelvic bone or the femur are affected [14]. Several interventions are generally required, as observed in our series and in other series previously published [10]. In long bones, osseous CE can occasionally be completely resected with severe sequels from the potential amputation of the limb [25]. When osseous CE affects the pelvis, prognosis may depend on whether the coxo-femoral or the sacroiliac joints are affected or not [26]. When these joints are affected, complete resection may not be possible or cause severe functional disability. In our series, two of the six patients with pelvic CE needed total limb amputation and two required an iliac osteotomy.

In spinal CE, the type of surgery will depend on the location and extent of the disease. The primary purpose of surgery is decompression and stabilization of the spinal cord. Decompression is generally performed by laminectomy [27,28]. In our series, the most common interventions for spinal osseous CE were laminectomy and surgical fixation.

As surgical treatment is challenging, in most cases a combined treatment based on surgery and long-term antiparasitic therapy is the therapy of choice. In our series, apart from surgery, 96% of patients received albendazole. Adjuvant medical therapy can be given preoperatively and/or postoperatively to control the disease locally and prevent systemic spread and recurrence. In other cases, antiparasitic drugs are the only therapeutic option when surgery is not possible or involves severe risk or sequels [29]. However, there is no consensus on how antiparasitic drugs must be administered, whether therapy must be administered in combination or not, or the duration of treatment. Most authors suggest that when surgery is not curative or osseous CE is inoperable, medical treatment must be lifelong to control cyst growth [19]. In our series, the duration of therapy in patients who received albendazole was long, with an average of six years, reaching in some cases 16 years of medical treatment.

Albendazole was the first therapeutic option, as recommended in the literature [30]. The role of praziquantel in CE has not been well defined and there is insufficient data to support a clear recommendation for the use of praziquantel in prolonged chemotherapy [31]. Praziquantel seems to have a synergistic effect by increasing albendazole plasma levels and there is some evidence to support a role for the use of praziquantel in combination with albendazole during surgery or percutaneous procedures [31–33]. Although there is no consensus on when combination therapy must be administered, it has been given when the patient does not respond to albendazole alone [27]. In our series, due to the severity of some cases, mainly those suffering from spinal CE, or those with inoperable or non-responding lesions, nearly 60% of our patients received combination therapy of albendazole plus praziquantel.

Five patients with spinal osseous CE were treated with albendazole plus praziquantel plus nitazoxanide. Nitazoxanide has shown to be active *in vitro* and *in vivo* against *E*. *multilocularis* and *E*. *granulosus*. Yet, its possible effectiveness seems to be better when it is given in combination with other antiparasitic drugs reporting best results for nitazoxanide in combination with albendazole [34–36]. Our data on osseous CE and disseminated CE have been published elsewhere. We found that the combination of nitazoxanide with albendazole +/- praziquantel is effective for disseminated CE involving soft tissue, muscle or viscera. However, this combination therapy was not effective for chronic and extensive osseous lesions [37].

Patients with osseous CE require long-term follow-up for potential recurrence and possible complications or sequels associated with surgery or antiparasitic therapy [10]. Because a curative therapy is rarely feasible, follow-up is lifelong. In our series, the average duration of follow-up exceeded 20 years, and during that time nearly 93% of patients relapsed. Surveillance must be based on clinical data and radiology. As recurrence cannot be confirmed by serology, it is not used for follow-up [4]. The most common sequels reported in our series were medullary syndrome and severe pain in spinal CE. In pelvic CE, limb amputations and other severe functional disabilities were described. In patients with rib CE and femur CE, the most frequent sequel was severe pain. This is consistent with the literature, where persistent pain, fractures, paraplegia in spine CE or even death due to progression of the disease have been reported as frequent sequels and complications of osseous CE [10].

A strength of this study is that it includes patients with long-term follow-up. This offers a global vision of the evolution of osseous CE over time. However, the low incidence of osseous CE and the heterogeneity of cyst sites make it difficult to establish a diagnostic or therapeutic protocol based on our results. One possible limitation of this series is that it may provide information only about those most complicated cases of osseous CE, which are more frequently refereed to reference units.

### Conclusions

This article reports experience with the management of osseous CE in a referral unit of tropical and parasitic diseases in Spain. Fortunately, the incidence of osseous CE is low, but its diagnosis and management is highly challenging. Diagnosis must be based on a combination of indicators based on clinical symptoms, radiological findings and serological results. In most cases, osseous CE must be considered a chronic disease, since complete surgical resection is unlikely and antiparasitic drugs are rarely curative. The absence of protocols for the management of this disease and its low prevalence force physicians to approach the disease on a case-by-case basis. Additionally, osseous CE requires long-term follow-up for recurrence and possible sequels.

## Supporting information

S1 Table Locations of osseous cystic echinococcosis based on a single or multiple bones affected. (DOCX)
