# Supplementary material for: Osseous cystic echinococcosis: A case series study at a referral unit in Spain
Source: PLoS Negl Trop Dis. 2019 Feb 19;13(2):e0007006. doi: 10.1371/journal.pntd.0007006 (PMC6396934; doi:10.1371/journal.pntd.0007006)
Supplement: S1 Table — (DOCX) [file pntd.0007006.s001.docx]

S1 Table. Locations of osseous cystic echinococcosis based on a single or multiple bones affected.

| **Skeletal area: number of patients** | **Location: number of patients** | **Associated with another bone site: number of patients** |
| --- | --- | --- |
| Axial skeleton: 25 | Vertebrae: 17 | Vertebrae only: 6 |
|  |  | Vertebrae + Ribs: 8 |
|  |  | Vertebrae + Pelvis: 2 |
|  |  | Vertebrae + Ribs + Sternum: 1 |
|  | Ribs only: 2 | |
|  | Pelvis: 6 | Pelvis only: 2 |
|  |  | Pelvis + Femur: 2 |
|  |  | Pelvis + Femur + Tibia: 2 |
| Appendicular skeleton: 1 | Femur only: 1 | |
| Other: 1 | Scapula only: 1 | |

Total: 44 locations in 27 patients
